# Collective behavior emerges from genetically controlled simple behavioral motifs in zebrafish

**DOI:** 10.1126/sciadv.abi7460

**Published:** 2021-10-06

**Authors:** Roy Harpaz, Ariel C. Aspiras, Sydney Chambule, Sierra Tseng, Marie-Abèle Bind, Florian Engert, Mark C. Fishman, Armin Bahl

**Affiliations:** 1 Department of Molecular and Cellular Biology, Harvard University, Cambridge, MA 02138, USA.; 2 Center for Brain Science, Harvard University, Cambridge, MA 02138, USA.; 3 Harvard Department of Stem Cell and Regenerative Biology, Harvard University, Cambridge, MA 02138, USA.; 4 Biostatistics Center, Massachusetts General Hospital, Boston, MA 02114, USA.; 5 Centre for the Advanced Study of Collective Behaviour, University of Konstanz, Konstanz 78464, Germany.

## Abstract

It is not understood how changes in the genetic makeup of individuals alter the behavior of groups of animals. Here, we find that, even at early larval stages, zebrafish regulate their proximity and alignment with each other. Two simple visual responses, one that measures relative visual field occupancy and one that accounts for global visual motion, suffice to account for the group behavior that emerges. Mutations in genes known to affect social behavior in humans perturb these simple reflexes in individual larval zebrafish and change their emergent collective behaviors in the predicted fashion. Model simulations show that changes in these two responses in individual mutant animals predict well the distinctive collective patterns that emerge in a group. Hence, group behaviors reflect in part genetically defined primitive sensorimotor “motifs,” which are evident even in young larvae.

## Teaser

Emergent social behavior in larval zebrafish is altered by targeted gene mutations.

## INTRODUCTION

Collective movements of animal groups are adaptive (*1*) because they provide protection from predation (*2*, *3*), improve foraging (*4*–*6*), and enhance energy utilization (*7*, *8*). Extensive evidence has shown that such group behaviors can emerge from local interactions among individuals (*9*–*20*). However, it is not known how the genetic makeup of animals affects the sensorimotor algorithms implemented by individual animals that give rise to emergent patterns of collective behavior.

Zebrafish display a variety of distinct group behaviors, including shoaling, where individuals swim in proximity, and schooling, where all members of the group move in the same direction. To achieve such synchronized movements in groups, individual members need to assess certain properties of their near neighbors, including their speed, distance, and orientation; they also need to rapidly respond to these features and execute the appropriate motor commands. The ability to perform such socially relevant sensorimotor transformations, and thereby the ability to form groups, varies among different genetic backgrounds (*21*–*24*) and is modified by hunger (*25*–*27*) and innate interindividual differences (*28*–*31*). Furthermore, whereas inputs from several sensory modalities such as lateral line mechanoreception (*32*, *33*), olfaction (*34*), and vision likely play a role in this process, vision is critical to certain attributes, such as the rapidity of turning responses, the necessary integration of distal cues, and the precision of the alignment responses (*9*, *10*, *24*). Mapping the algorithmic rules and neurophysiology underlying collective behaviors can be more readily accomplished in larval zebrafish when the brain is transparent and circuits are simpler than in adults (*35*). However, while reflexive responses to stimuli emanating from conspecifics have been described in various contexts (*32*, *33*), there has been little evidence of robust shoaling- or schooling-like behavior in zebrafish larvae younger than ~10 days post fertilization (dpf) (*13*, *30*, *36*).

Here, we identify two visual reflexes that are present from 7 dpf. We believe that the implementation of these reflexes leads to emergent patterns of the groups as fish mature. First, young larvae appear to repel away from regions of high visual clutter, leading to a dispersal of the group. At later developmental stages, this dispersal reflex shifts to attraction and hence leads to the observed aggregation behaviors. Second, larvae are known to move along with external motion stimuli, a well-described behavior known as the optomotor response (OMR) (*37*–*39*). We hypothesize that individuals swimming within a group can cue in on their neighbors’ relative motion and that this reflex might help in improving mutual alignment. The combined developmental maturation of both reflexes can thus explain emergent shoaling and schooling behavior.

Notably, mutations in genes associated with autism and schizophrenia quantitatively alter these two visuomotor responses, and these changes seem to be predictive of the distinct emergent behaviors of groups of mutant fish. Thus, subtle alterations in simple behavioral motifs in the individual can account for complex emergent patterns of groups.

## RESULTS

### Visually driven aggregation and alignment in larval and juvenile zebrafish

**Fig. 1.**
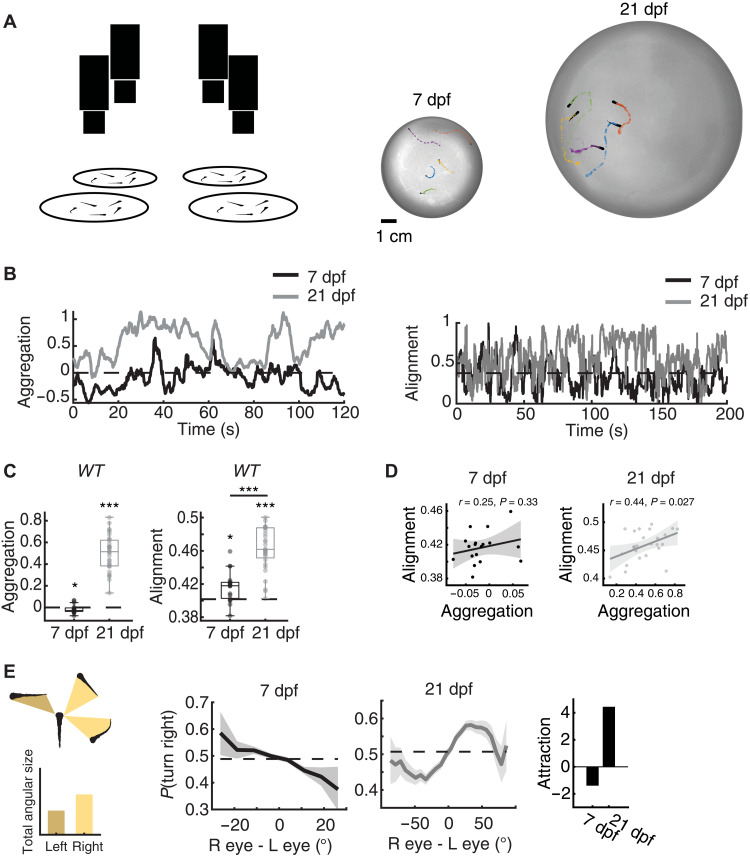
Aggregation in developing zebrafish. (**A**) Left: Groups of five fish were tested in circular arenas, while overhead cameras recorded their behaviors. Right: Position and body orientations of each fish were extracted from the movies. (**B**) Example traces of the aggregation index (left) and alignment index (right) (see Methods) of groups of 7- and 21-dpf wild-type fish. Dotted lines represent baseline dispersion and aggregation levels of shuffled control groups. At 21 dpf, fish show higher aggregation and alignment than 7-dpf fish. (**C**) Left: At 7 dpf, fish are less aggregated than shuffled control groups (*P*_Bootstrap_ ≈ 0.047, *N* = 18 groups; Cohen’s *d* = −0.49) (see Methods), while 21-dpf fish form tight groups (*P*_Bootstrap_ < 1/100,000; *N* = 25 groups, Cohen’s *d* = 2.5). Right: 7-dpf fish are more aligned than shuffled control groups (*P*_Bootstrap_ ≈ 0.0002, Cohen’s *d* = 0.8), and 21-dpf fish are more aligned than 7-dpf fish (*P*_Fisher_ < 1/100,000; Cohen’s *d* = 1.8). *P < 0.05, ***P < 0.0005. (**D**) Pearson’s correlation of alignment and aggregation in 7-dpf (left) and 21-dpf (right) fish. Uncertainty regions are based on pointwise 95% CIs of the linear regression model (Methods). (**E**) Effect of “visual clutter.” Left: We reconstruct the visual angle that each neighboring fish is expected to cast on the retina of a focal fish (see Methods). Middle: The difference between total angular area (or visual clutter) experienced by each eye modulates the probability to turn away (7 dpf) or toward (21 dpf) the more cluttered visual field. Bold lines represent turning probability calculated from left/right turning events recorded from all fish in 5° bins. Uncertainty regions are based on pointwise 95% CI of a fitted binomial distribution to the events in each bin. Right: The integral of the curves in the middle panels symmetrized such that repulsion from clutter is negative and attraction is positive.

We examined the behavior of wild-type larval zebrafish in groups of five, swimming together in a circular arena, where an overhead camera was used to monitor the position, orientation, and speed of each individual over extended time periods (see Fig. 1A and Methods). As animals mature from 7 to 21 dpf, we observed that they swim more closely to one another (Aggregation_7 dpf_ = −0.019 ± 0.04 [means ± SD], Aggregation_21 dpf_ = 0.476 ± 0.19, *P*_Fisher_ < 1/100,000), are more aligned (Alignment_7 dpf_ = 0.417 ± 0.018 [means ± SD], Alignment_21 dpf_ = 0.458 ± 0.027, *P*_Fisher_ < 1/100,000), and exhibit faster swim speeds (Speed_7 dpf_ = 0.34 ± 0.07 cm/s [means ± SD], Speed_21 dpf_ = 0.6 ± 0.17 cm/s, *P*_Fisher_ < 1/100,000) (Fig. 1, A to C, and fig. S1A). In addition, at 21 dpf, larvae exhibit a positive correlation between mutual alignment and aggregation (*r*_Pearson_ = 0.44, *P* = 0.027) (Fig. 1D), a phenomenon that is consistent with the plausible concept that conspecifics that swim closer to each other evoke stronger alignment responses than do more distant fish. Even at 7 dpf, groups already exhibit evidence of interactions. However, at this early age, the aggregation indices appear to be negative [mean Aggregation_7 dpf_ = −0.019, 95% bootstrap interval (BI): [−0.0362; −0.0004], two-sided *P*_bootstrap_ ≈ 0.047], indicating that young larvae may display mutual repulsion rather than attraction. Despite the resulting increase in interindividual distances, we observe that alignment is present at this early age (mean Alignment_7 dpf_= 0.417, 95% BI: [0.4086 0.4252], two-sided *P*_bootstrap_ ≈ 0.0002) and becomes stronger in older fish (Fig. 1C). This result also serves as a motivation to use higher-sensitivity assays to explore the mechanism by which fish align to global visual motion stimuli, as described later.

To explore the algorithmic basis of the transition from dispersed to aggregated groups with age, we examined the turning behavior of fish in response to the occupancy of their right and left visual fields, i.e., the retinal “clutter” generated by the presence of conspecifics swimming in the vicinity (Fig. 1E, left) (*11*, *13*). We observe that 7-dpf fish tend to turn away from the more highly cluttered area, whereas 21-dpf animals turn toward it [e.g., *p*(turn right) = 0.407, 95% confidence interval (CI): [0.339 0.479] for +25° clutter difference (more clutter to the right) at 7 dpf, whereas *p*(turn right) = 0.589, 95% CI: [0.580 0.597] for +25° at 21 dpf] (Fig. 1E). This simple visuomotor response to differences in retinal clutter leads to a dispersal (7 dpf) or aggregation (21 dpf) phenotype.

**Fig. 2.**
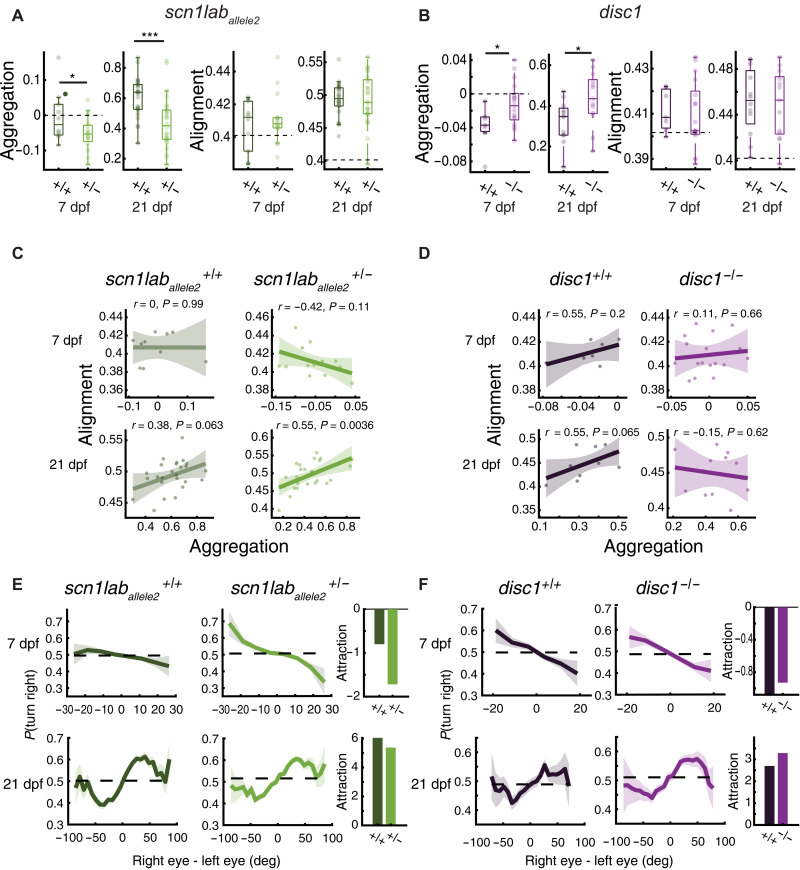
Single-gene mutations affect aggregation and alignment of developing zebrafish. +/+, +/− , and –/– refer to sibling-controlled wild-type and mutant fish. (**A**) Left: At 7 dpf, *scn1lab^+/−^* fish are more dispersed than wild-type siblings (*P*_Fisher_ ≈ 0.036, *N*_+/+_ = 10, *N*_+/−_ = 16 groups, Cohen’s *d* = −0.8). Dashed lines represent values of shuffled groups. At 21 dpf, fish are more aggregated. *Scn1lab^+/−^* aggregate less than *scn1lab^+/+^* (*P*_Fisher_ ≈ 0.0001; *N*_+/+_ = 25, *N_+/−_* = 26 groups, Cohen’s *d* = −1.09). Right: Group alignment increases with age; however, we could not detect an effect of the *scn1lab* mutation (*P*_*7* Fisher_ = 0.26, *P*_*21* Fisher_ = 0.47). *P < 0.05, ***P < 0.0005. (**B**) Left: At 7 dpf, *disc1^−/−^* are less dispersed than wild-type siblings (*P*_Fisher_ ≈ 0.0128; *N*_+/+_ = 7, *N*_−/−_ = 17 groups, Cohen’s *d* = 1.04). At 21 dpf, *disc1^−/−^* show more aggregation compared to wild-type siblings (*P*_Fisher_ ≈ 0.0129; *N*_+/+_ = 12, *N*_−/−_ = 13 groups, Cohen’s *d* = 0.95). Right: We could not detect effect of the *disc1* mutation on alignment (*P*_*7* Fisher_ = 0.33, *P*_21 Fisher_ = 0.40). (**C**) Pearson’s correlation of alignment and aggregation in *scn1lab_allele2_* 7-dpf (top) and 21-dpf (bottom) fish. Positive correlation for 21-dpf *scn1lab_allele2_^+/−^* fish. Uncertainty regions are the pointwise 95% CI of the linear regression model. (**D**) We could not detect correlation for *disc1*. (**E**) Left: *scn1lab_allele2_^+/−^* turn away more from visual clutter at 7 dpf (top) and turn toward clutter less at 21 dpf (bottom). Right: Integral of the curves symmetrized. Repulsion is negative. Attraction is positive. (**F**) Same as in (E) but for *disc1*. Mutants show a flattening of the 7-dpf repulsion curve (top) and an enhancement in 21-dpf attraction (bottom). Bold lines in (E) and (F) represent turning probability calculated from left/right turns of all fish in 5° bins; uncertainty regions are the pointwise 95% CI of a fitted binomial distribution to the events in each bin.

We next tested whether perturbations by targeted genetic mutations cause specific changes in the clutter avoidance curves (Fig. 1E), thereby generating quantitative changes in aggregation indices and the associated shoaling phenotype in mutant animals. To that end, we selected fish with mutations in two genes that have been shown to generate specific social phenotypes in adults (*24*). The first gene, *scn1lab*, codes for a sodium channel. Its mutation is associated with Dravet’s syndrome in humans and causes more scattered group behaviors in adult zebrafish. We evaluated heterozygous fish of two alleles (*scn1lab_allele1_* and *scn1lab_allele2_*), because homozygous mutations are early lethal. The second gene, *disrupted-in-schizophrenia* (*disc1*), encodes a scaffolding protein associated with schizophrenia in humans; zebrafish with a homozygous deficiency in *disc1* display increased group cohesion as adults (*24*). We find that larvae with mutations in both genes behave differently from wild-type fish in the group swimming assay in a manner that is consistent with the differences described in mutant versus wild-type adults (*24*). At 7 dpf, both *scn1lab*^+/−^ alleles show an increase in dispersal when compared to their wild-type siblings, which manifests as a reduced aggregation index (Aggregation_7 *scn*1*lab* + /−_ = −0.052 ± 0.047 [means ± SD], Aggregation_7 *scn*1*lab* + /+_ = −0.005 ± 0.073, one-sided *P*_Fisher_ ≈ 0.035) (Fig. 2A and fig. S1B). At 21 dpf, both *scn1lab^+/−^* and *scn1lab^+/+^* switch from dispersal to aggregation, but mutant fish are still less aggregated than their sibling controls (Aggregation_21 *scn*1*lab* + /−_ = 0.44 ± 0.17 [means ± SD], Aggregation_21 *scn*1*lab* + /+_ = 0.6 ± 0.13, one-sided *P*_Fisher_ ≈ 0.0001) (Fig. 2A and fig. S1B), which is in line with the reduced aggregation phenotype reported in adults (*24*). In contrast, 7-dpf *disc1*^−/−^ fish show an increase in aggregation relative to their *disc1^+/+^* sibling controls (Aggregation_7 *disc* − /−_ = −0.012 ± 0.026 [means ± SD], Aggregation_7 *disc* + /+_ = −0.038 ± 0.024, one-sided *P*_Fisher_ ≈ 0.0128), an effect that is also present at 21 dpf (Aggregation_21 *disc* − /−_ = 0.44 ± 0.13 [means ± SD], Aggregation_21 *disc* + /+_ = 0.32 ± 0.1, one-sided *P*_Fisher_ ≈ 0.0129) (Fig. 2B).

We next examined whether the responses of the mutant fish to retinal clutter are concordant with the observed aggregation indices: Compared to wild-type siblings, we observed that *scn1lab*^+/−^ fish have an enhanced tendency to turn away from high clutter at 7 dpf (symmetrized area under the curve sAUC_7 *scn1lab+/−*_ = −1.7, sAUC_7 *scn1lab+/+*_ = −0.8), and they may have a slightly reduced tendency to turn toward high clutter at 21 dpf (sAUC_21 *scn1lab+/*−_ = 5.3, sAUC_21 *scn1lab+/+*_ = 6.0) (Fig. 2E). The *disc1*^−/−^ mutation, on the other hand, displays a slight “flattening” at the edges of the clutter response curve at 7 dpf (sAUC_7 disc−/−_ = −0.93, sAUC_7 disc+/+_ = −1.10), suggesting less repulsion, and a similarly small enhancement of the tendency to turn toward high visual clutter at 21 dpf (sAUC_21 disc−/−_ = 3.3, sAUC_21 disc+/+_ = 2.7) (Fig. 2F). These trends are both qualitatively in line with the increase in aggregation indices at both ages (Fig. 2B). However, uncovering the precise relationship between the turning curves and aggregation indices requires a more detailed and quantitative analysis, as we describe in our collective behavior models below.

Both wild-type and mutant fish show enhanced mutual alignment by 21 dpf compared to 7 dpf (Fig. 2, A and B). However, our data did not allow us to report an alignment difference between mutant and wild-type animals at any age or strain (*P*_Fisher_ ≈ 0.26 and 0.47 for 7- and 21-dpf *scn1lab*; *P*_Fisher_ ≈ 0.47 and 0.33 for 7- and 21-dpf *disc1*).

We observe a positive correlation of alignment with aggregation in *scn1lab^+/−^* 21-dpf mutants in both alleles (*r*_Pearson_^*allele*2^ = 0.55, *r*_Pearson_^*allele*1^ = 0.65) (Fig. 2C and fig. S1D), whereas in 21-dpf *disc1^−/−^* animals, no correlation can be inferred from the data (Fig. 2D). This observation suggests that mutations might cause subtle changes in alignment dependent on proximity, which we explore further using a more targeted approach to extract alignment phenotypes, as described below in the OMR experiments.

In summary, the tendency of 7-dpf animals to move away from visual clutter and their enhanced alignment indices indicate that conspecific fish, even at this young age, interact with each other. This interaction precedes the well-described tendency to move toward clutter by 21 dpf (*13*). Notably, we could estimate the effects of mutations associated with human social disorders on behavior as early as 7 dpf, and these effects become more pronounced as animals mature.

### Mutant larval zebrafish show specific changes in their ability to align with motion

**Fig. 3.**
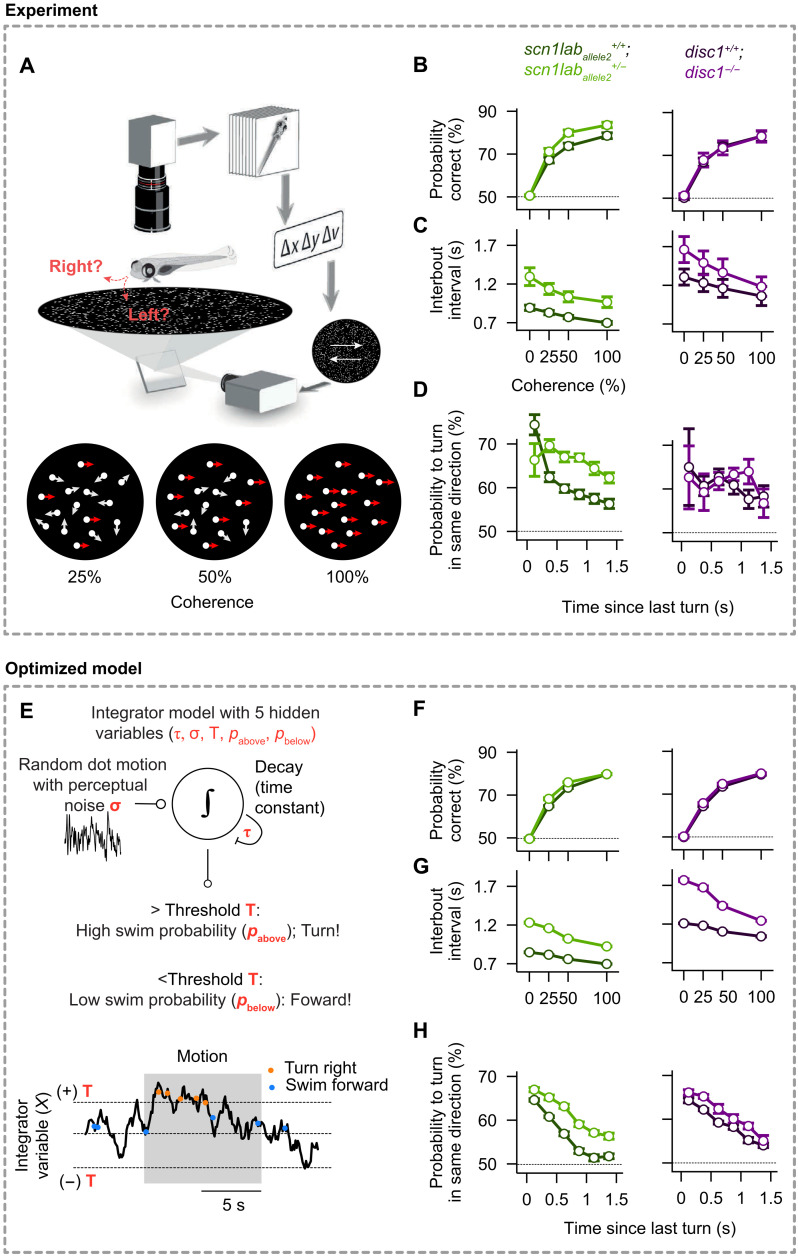
Mutant 7-dpf larval zebrafish display differential integration and alignment phenotypes, which can be quantitatively captured by a simple integrator model. (**A**) Experimental setup [adapted from (*39*)]. A single larval zebrafish swims freely on top of a projected cloud of randomly moving dots. Dots move continuously at different coherence levels either to the right or left relative to the body orientation of the animal (movie S1). (**B**) Probability to correctly align with the coherent motion stimulus as a function of coherence strength. *Scn1lab_allele2_^+/−^* mutant fish (bright green) show improved performance relative to *scn1lab_allele2_^+/+^* wild-type sibling controls (dark green). Our data did not allow us to report a difference in performance of the *disc1* mutant (magenta) compared to sibling controls (black). (**C**) Interbout interval as a function of coherence. Values are elevated for both mutants relative to wild-type sibling controls. (**D**) Tendency to turn in the same direction as a function of the time since the last bout during randomly flickering 0% coherence stimulation. Responses are elevated for the *scn1lab_allele2_* mutant relative to wild-type sibling controls. (**E**) Integrator model with decision threshold (T), perceptual noise (σ), leak time constant (τ), and probabilities to make a turn or swim forward (*p*_above_ and *p*_below_, depending on whether the integrated value is above or below the threshold). (**F** to **H**) Optimized model results, analyzed and displayed as in (B) to (D). The model accurately captures the behavioral features of both wild-type and mutant larvae. *N* = 44, 36, 21, and 16 individually tested fish for genotypes *scn1lab_allele2_^+/+^*, *scn1lab_allele2_^+/−^*, *disc1^+/+^*, and *disc1^−/−^*, respectively, in (B) to (D). *N* = 12 models (different optimization repeats) for each genotype in (F) to (H). Error bars in (B) to (D) and (F) to (H) are ± SEM.

Alignment among adult fish can arise from attraction and repulsion alone, and in many cases, it has been shown that an explicit alignment process is not required (*9*, *10*, *12*). However, explicit alignment reflexes, in particular to moving cues, are known to exist in larval fish (*37*–*39*), and we therefore explored this possibility by analyzing such explicit responses to motion cues in mutant and wild-type strains. Larval zebrafish turn in response to motion of dots in their visual fields, which can be quantified by a coherent dot–based optomotor response (OMR) assay (*39*, *40*). We, therefore, presented 7-dpf free-swimming individuals with clouds of flickering small dots that drifted either to the right or left, relative to their body orientation (Fig. 3A and movie S1). The limited lifetime and partial global coherence of these dots make it more challenging to identify motion direction, such that fish have to temporally integrate information to make appropriate swimming decisions (*39*, *40*). These decisions can be quantified as a function of dot coherence, the proportion of swims following the direction of the motion stimulus (“probability correct”), and the time of quiescence between consecutive swims (“interbout interval”) (Fig. 3, B and C). We also estimated the probability of swimming in the same direction for consecutive bouts, even when not stimulated by motion drift, to assess the tendency of larval zebrafish to repeat the same motor action over extended periods of time (Fig. 3D and fig. S2, B and C) (*41*).

We find that, compared to wild-type siblings, fish of both *scn1lab^+/−^* alleles have an increased probability of responding correctly as a function of coherence (two-sided *P*_Fisher_ ≈ 0.95,0.045,0.001, and 0.001, for coherences of 0, 25, 50, and 100%) and that they respond with longer delays as seen by increased interbout intervals (two-sided *P*_Fisher_ <1/100,000, for all coherences) (Fig. 3, B and C, and fig. S2). They also show an increased probability of turning in the same direction for consecutive bouts (two-sided *P*_Fisher_ ≈ 0.08,0.0001,0.0001,0.00001,0.00001, and 0.0004 for time delays of 0.125 to 1.375 s) (Fig. 3D). The *disc1*^−/−^ mutants, on the other hand, differ only in having longer interbout intervals than do their wild-type siblings (two-sided *P*_Fisher_ ≈ 0.08,0.18,0.35, and 0.51, for coherences of 0, 25, 50, and 100%) (Fig. 3C). Turning distributions of mutant animals and sibling controls indicate an increase in turn angle compared to controls, for both *scn1lab* alleles for the highest coherence level (two-sided *P*_Fisher_ ≈ 0.0001,0.8, and 0.0002 for *scn1lab_allele2_*, *disc1*, and *scn1lab_allele1_*) (fig. S2C). Hence, mutations in both genes cause subtle differences in the animals’ ability to integrate information over time and to align with motion drift in their environment.

### Drift-diffusion model for motion integration to explain alignment with motion cues

We have previously shown that the responses of individual larval zebrafish to coherent dot motion can be well described by the computational framework of a “drift-diffusion model” (DDM) (Fig. 3E) (*42*, *43*). This model uses only five parameters: the internal noise (σ), integration and decay time constants (τ), decision thresholds (**T**), and two swimming probabilities (*p*_below_ and *p*_above_; see Methods) to fully describe the behavior. Notably, none of these parameters can be measured directly through experiments. We therefore resorted to a multiobjective fitting approach, which allowed us to automatically extract those values and systematically explore their variation due to the mutations (see figs. S3 and S4 and Methods). We show that this strategy quantitatively captures subtle behavioral features across the different mutants. For example, we find that, when compared to the respective sibling control animals, the threshold variable (T) decreases in *scn1lab* mutant fish, whereas it increases for the *disc1* mutant (fig. S4B). Thus, modeling wild-type and mutant fish behavior exclusively based on the DDM with these extracted variables allowed us to test whether this framework is sufficient to explain the behavioral results.

We find that the model predicts most of the experimental data (Fig. 3, B to D and F to H, and fig. S3, B, C, E, and F), which suggests that the DDM provides an adequate framework to quantitatively describe alignment behavior in groups and that it is capable of reliably extracting hidden integration and decision-making variables in mutant animals. Furthermore, a quantitative evaluation of these behavioral variables allows us to make specific predictions about corresponding neural circuit changes in mutant animals (*39*, *40*), and they can also provide the critical substrate for model simulations of fish in more complex scenarios (see below).

### Models based on two simple reflexes explain emergent collective behavior

**Fig. 4.**
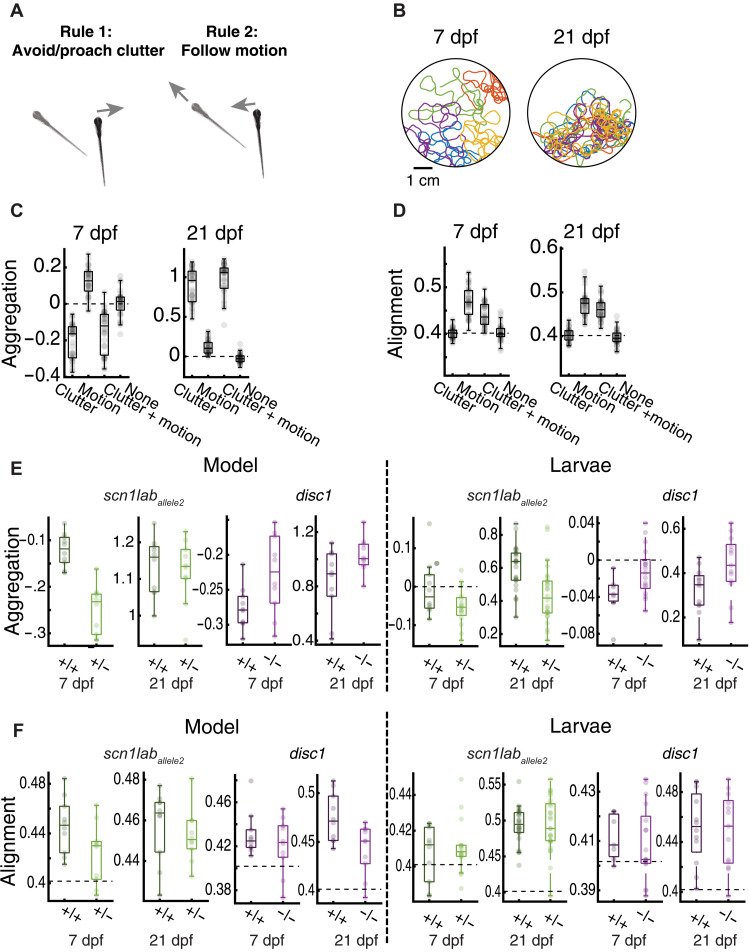
Simple visuomotor reflexes qualitatively predict emergent group behavior across genotypes and development. (**A**) Schematic of the model using only two simple algorithmic rules: First, fish are repelled or attracted to visual clutter (see Fig. 1E). Second, fish use global motion cues for turning decisions (see Fig. 2, A and E). Both cues are integrated over time and evaluated according to our integrator model (Fig. 2E). Model parameters for the clutter response strength are directly extracted from group swimming experiments (Fig. 2, E and F). Model parameters for the integration and decision-making process are taken from our multiobjective parameter fitting results (fig. S4B). The model is hence almost fully constrained by experimental data. (**B**) Example trajectories of simulated wild type 7 and 21 dpf based on the rules shown in (A). (**C** and **D**) Aggregation and alignment for wild-type simulations for all possible rule combinations (neither rule, only motion, only clutter, or both rules) for 7- and 21-dpf model fish. Alignment does not emerge with attraction alone; it additionally requires animals to perform motion integration. Conversely, motion integration induces some level of aggregation. Parameters for wild-type animals are the same as for the sibling controls for all tested mutant lines. (**E** and **F**) Aggregation and alignment for 7- and 21-dpf mutant model fish with respective sibling controls (left) and the corresponding data of real fish (right; same data as in Fig. 2). Our model can qualitatively predict all group behavior phenotypes across ages as found in our experimental group assay (Fig. 2, A and B). *N* = 36 model simulations (each model uses different parameter sets, following our repeated model optimization; fig. S4B) in (C) and (D) and *N* = 12 model simulations for each genotype in (E) and (F). Experimental data in (E) and (F) are the same as in Fig. 2 (A and B).

We tested whether the two basic reflexes, i.e., the “clutter response” (Fig. 1E) and the coherent moving dot response (cdOMR) (Fig. 3A), are sufficient, when applied to individual fish, to explain the different emergent behaviors of the groups. To that end, we simulated groups of five virtual fish swimming in a circular arena, in which each individual agent follows only the computations predicted by our two assays (Fig. 4A). The “clutter response computation” is the measurement of the clutter projected by the four conspecifics onto the left and right eyes. The “moving dot computation” measures the retinal motion component generated by all other fish (see Methods). Both signals are integrated over time and compared to the threshold, allowing the model to make decisions about whether to move forward or make turns, as described by the DDM model (see Fig. 3E and Methods). Using these two computations, we simulated swim trajectories of groups of 7-dpf and 21-dpf “virtual” fish (Fig. 4B and movies S2 and S3) and extracted aggregation and alignment indices, as done for groups of real fish (Fig. 1). Simulations corresponding to the different experimentally obtained variables and response curves revealed that the model produces results that qualitatively match the experimental findings: 7-dpf wild-type virtual larvae show a tendency to repel each other (with slightly negative aggregation indices), whereas 21-dpf virtual animals show strong aggregation behavior (Aggregation_7 dpf_ = − 0.15 ± 0.11, Aggregation_21 dpf_ = 0.98 ± 0.2) (Fig. 4C). Alignment indices increase from 7 to 21 dpf (Alignement_7 dpf_ = 0.44 ± 0.025, Alignment_21 dpf_ = 0.46 ± 0.023), as observed in the experimental results (Fig. 4D). Because we used the same cdOMR variables for 7- and 21-dpf simulations, this improvement in alignment is likely a consequence of the enhanced aggregation values in older animals, which leads to a more pronounced effect of visual motion cues and consequently stronger alignment (Fig. 1D). To further, and explicitly, probe the interdependence of aggregation and alignment, we asked how well each rule by itself predicts aggregation and alignment indices, respectively (Figs. 4C and 4D) and how they interact when combined. We find that aggregation indices are, as expected, dominated by the clutter response rule (although the addition of the motion response rule slightly enhances aggregation). Alignment indices, on the other hand, which depend predominantly on the motion response rule, are modulated and brought into far better agreement with observed data when the clutter response is added. Thus, the combination of the two attributes, clutter response and dot motion response, predicts that the reduced aggregation of 7-dpf fish will lead to weaker alignment, and the strong aggregation in 21-dpf animals will lead to stronger alignment, both results that are concordant with the experimental data.

We next used this modeling framework to simulate mutant animals, by combining both clutter and motion computations and using the model parameters that we extracted from our experimental assays for each genotype (Fig. 2, E and F, and figs. S1E and S4B). Here we find, also in agreement with the experimental data, that the tendency of 7-dpf wild-type virtual larvae to repel each other is enhanced in *scn1lab*^+/−^ mutant fish and diminished in *disc1*^−/−^ animals (Fig. 4E). This phenotype carries robustly into 21-dpf animals, where *disc1*^−/−^ zebrafish show an increased aggregation phenotype compared to wild-type virtual siblings. The success of this minimal model in qualitatively reproducing the experimental results suggests that, at least in the larval animal, genetic effects upon just the two visual responses suffice to explain core attributes of the emergent behavior of the group.

## DISCUSSION

Here, we find that even young larval zebrafish interact with each other and that their swimming dynamics are well predicted by two visual responses: the retinal clutter response and the OMR. These two visuomotor assays explore the tendency of fish to attract to each other and to align their swimming direction with motion cues, respectively, both key attributes of collective behaviors. We show that mutations in genes associated with autism and schizophrenia alter these two visual responses in subtle ways and that these changes are qualitatively predictive of emergent mutant shoaling and whole field motion alignment phenotypes. The effects of the two mutations on group dynamics can be detected in fish as young as 7 dpf, and they are qualitatively similar to the effects of the same mutations in groups of adult fish (*24*). Specifically, mutation of the *scn1lab* gene, the ortholog of which is associated with Dravet’s syndrome of childhood epilepsy and autism, causes fish to swim in a more dispersed fashion, and a mutation of *disc1*, associated with schizophrenia, causes fish to huddle more closely.

Larvae at 7 dpf do not aggregate into shoals or seek the vicinity of other fish (*13*, *30*, *36*), so responses to conspecific stimulation observed at this early age (*32*, *33*) have been assumed to be unrelated to shoaling or schooling behavior. Here, we find that larval zebrafish repel, rather than attract, each other at this young age, which leads to a distinct global dispersion—or negative aggregation—phenotype. This repulsion phenotype switches to attraction with age, so that by 21 dpf, the animals tend to form more familiar aggregates and shoals (*13*, *36*). Because 7-dpf larvae are not very motile and tend to live in protected areas with little water flow, this repulsion might assure them of sufficient oxygenation from relatively unstirred surroundings (*44*), and it might help them avoid frequent collisions in the cramped quarters typical for densely populated clutches. The switch to aggregation at 21 dpf likely helps keep groups of older animals together when they start exploring larger areas of their environment and when they begin swimming over longer distances. We also note an alignment tendency between fish, weak at 7 dpf and stronger at 21 dpf, which we cannot fully explain by the clutter response, and which we find better predicted by turning responses of the individual fish to fields of moving dots. In adult fish, it has been shown that alignment can emerge purely on the basis of attraction and repulsion algorithms (*9*, *10*, *12*), although some have also speculated a need for specific alignment forces between fish (*14*, *16*).

The mutations do not appear to affect alignment among the fish in free-swimming groups. However, 21-dpf *scn1lab^+/−^* mutant fish did show a correlation of alignment with proximity (Fig. 2C), suggesting that these mutants might be more responsive to nearby moving stimuli. Adult *scn1lab^+/−^* mutant fish also exhibit enhanced alignment when swimming in close proximity (*24*). In the coherent dot assay, *scn1lab^+/−^* mutant fish align more accurately with moving dots than do their wild-type siblings. Thus, the cdOMR can serve as a powerful tool to uncover subtle differences in responses to motion in larvae.

To combine both visuomotor transformations into a unified model, we extracted the interocular retinal clutter difference, calculated an induced turning weight (see fig. S5 on the computation of turning weight), and fed that turning weight as an additional gain into the DDM as detailed for the cdOMR (Fig. 3E). We have chosen this particular implementation based on the physiological and anatomical evidence for a multimodal hindbrain integrator circuit. A particular cluster of neurons in the ventral hindbrain of larval zebrafish has been shown to receive multimodal information relevant to turning responses, including luminance information (*45*) and dot motion OMR (*39*, *40*). These data make it plausible that integration of visual clutter and motion information also occurs in the same brain area.

Our model, which is based solely on these two simple visuomotor transformations, can account for a large fraction of the complex collective interactions that occur in groups. The model’s simplicity and its applicability even to 7-dpf larvae with their relatively simple and accessible brains make it a practical entry point to dissect the cellular nature of the algorithms that drive collective behaviors. Although fish have many sensory inputs that likely contribute to group behaviors, zebrafish are highly visual (*39*, *41*, *46*), suggesting that visual drives likely play a dominant role. Other sensory modalities, such as somatosensation through the lateral line (*32*, *33*) and olfaction (*26*, *34*), undoubtedly play roles in modulating social interactions, as might currently less decipherable elements such as “internal state” (*25*–*27*) and “personality” (*28*–*31*).

The specific genetic perturbations that we have studied are in genes related to human psychiatric disorders. The human *SCN1A* gene (the ortholog of the zebrafish *scn1lab* gene) is associated with Dravet’s syndrome (where patients have epilepsy and developmental disorders including autism), and *DISC1* is associated with schizophrenia. Atypical visual reflexes, including the optokinetic response, have been noted in both autism and schizophrenia (*47*), and there is evidence for linkage between such perceptual differences and social abnormalities in autism (*48*). Perhaps reduction of complex behaviors to simple underlying reflexive motifs may help to characterize complex disorders. Quantitative characterization of changes in these reflexes in mutant zebrafish facilitates analysis of the underlying cellular defects and enables screens for therapeutics (*49*).

## METHODS

### Zebrafish

To generate larvae for sibling-controlled experiments, heterozygous fish were incrossed. For *scn1lab* experiments, the *scn1lab^+/−^* fish were crossed with AB wild type. Clutches were raised in small groups (20 to 30) in 15-cm petri dishes with fish facility water in 14-hour light, 10-hour dark cycle at constant 28°C. At 4 dpf, larvae were fed rotifers or paramecia daily with 50% water change. Behavior experiments were done at 7 and 21 dpf. All experiments followed protocols approved by the Harvard Institutional Animal Care and Use Committee.

### Group assay

We used custom-designed experimental arenas of different sizes: diameter of 6.5 and 12.6 cm (for groups of 7- and 21-dpf fish) and height of 1 cm made of ^1^/_16_ inch (~1.59 mm) polyethylene terephthalate glycol plastic (PETG). Arenas had a flat bottom and curved walls to encourage fish to swim away from the walls and were sandblasted to prevent reflections. Every experimental arena was filmed using an overhead camera and lit from below using infrared light (same as in the dot motion assay). Images were acquired at ~39 fps and were segmented online to separate fish images from the background. The segmented images were then analyzed offline to extract continuous tracks of the fish [see (*50*) for details of segmentation and tracking algorithms]. All acquisition and online segmentation were performed using custom-designed software written in MATLAB.

### Individual and group properties of free-swimming fish

We used the extracted center of mass position of every fish ($\text{fish}\;i;{\vec{x}}_{i}$) to calculate the velocity of the fish $\vec{v_{i}}(t)=\vec{[x_{i}}(t+\mathrm{dt})-\vec{x_{i}}(t-\mathrm{dt})]/2\mathrm{dt}$, where *dt* is 1 frame or 0.025 s. The speed of the fish is then $S_{i}(t)=|\vec{v_{i}}(t)|$, and the direction of motion is $\vec{d_{i}}(t)=\vec{v_{i}}(t)/|\vec{v_{i}}(t)|$.

For the group, we calculate a normalized measure of group aggregation: Aggregation = − log (*NN*_1_/*NN*_1_^shuffled^), where *NN*_1_ is the average nearest neighbor distance. *NN*_1_^shuffled^ is the same distance calculated from control groups created by shuffling fish between groups such that all fish in a shuffled group were chosen from different real groups. Positive aggregation values mean that real groups are more aggregated than shuffled controls, and 0 means aggregation occurred at random. Group alignment was defined as $\text{alignment}(t)=|\sum_{i}^{N}\vec{d_{i}}(t)|/N$, where *N* is the number of fish in the group, and alignment value is bounded between 0, all fish are pointing in different directions, and 1, all fish swim in the same direction.

### Estimating visual occupancy using ray casting

To estimate the visual angle that each neighbor in the group cast on the eye of a focal fish (fish *i*), we cast 1000 rays from each eye spanning 165° from the direction of motion toward the back of the fish, leaving a total of 30° of blind angle behind the fish. This amounts to an angular resolution of ~0.165° per line. We then detected all pixel values representing fish in the paths of the rays and calculated the visual angle occupied by each fish and the total visual angle experienced by each eye (Fig. 1E).

### Statistical analysis

We compared groups’ aggregation and alignment to the estimated “baseline” level obtained from shuffled groups (Fig. 1C) (see below), using a bootstrapping procedure (*51*). We estimated the sampling distribution based on the experimental sample by sampling with replacement *N* groups of five fish from the data 100,000 times. We constructed 95% BIs for the average statistic of these groups (i.e., alignment or aggregation) and also reported the associated *P*_bootstrap_ by inverting these intervals.

We also compared group aggregation and alignment between wild-type 7- and 21-dpf zebrafish. Here, we used groups of five zebrafish, which we denote by “j.” Let A_j_ denote the age of group j, where A_j_ = 0 for a 7-dpf group and A_j_ = 1 for a 21-dpf group, and assume a completely randomized assignment of the group age status. For each group j, we measure an outcome Y_j_, such as group aggregation or group alignment. We denote Y_j_(A_j_ = 0) the potential outcome had group j been 7 dpf and Y_j_(A_j_ = 1) the potential outcome had group j been 21 dpf. We test the sharp null hypothesis stating that, for each group j, the potential outcomes are equal, that is, Y_j_(A_j_ = 0) = Y_j_(A_j_ = 1). We choose Welch’s statistic (T_Welch_) as our test statistic. Following the procedure initially described in (*52*) and recently by various statisticians (*53*–*55*), we calculated two-sided Fisher *P* values (or approximated ones based on 100,000 randomized allocations).

To estimate the turning response probability (Figs. 1E and 2, E and F), we calculated the proportion of right turns out of all turns (left + right turns) recorded for a given difference in clutter between the left and right visual field (discretized into 5° bins). Because the number of observation in each bin was too large for calculating the exact binomial, the CI around this point estimate was calculated using the normal approximation of the binomial distribution: $p\pm z_{\pm 1.96}\cdot \sqrt{\frac{p\cdot (1-p)}{n}}$, where *p* is *p*(turn right), *z*_±1.96_ are the *z* scores defining the upper and lower bound containing 95% of a standard normal distribution, and *n* is the number of turns in a given bin. This estimation was performed independently for each bin, and the uncertainty region is shown as a continuous surface within the plots by connecting the edges of the individual estimates.

CIs for linear regression models (Figs. 1D and 2, C and D) were calculated as follows: ${\text{CI}}_{i}=y_{i}\prime \pm t_{\left(0.95,n-2\right)}\cdot S_{\text{es}t_{y}}\sqrt{\frac{1}{n}+\frac{{\left(x_{i}-\bar{x}\right)}^{2}}{\Sigma_{i}{\left(x_{i}-\bar{x}\right)}^{2}}}$where *y_i_*′ is the regression value at point *x_i_*, *t*_(0.95, *n* − 2)_ denote the upper and lower bounds comprising 95% of a Student’s *t* distribution with *n* − 2 df, and $S_{\text{es}t_{y}}=\sqrt{\frac{\Sigma_{i}{\left(y_{i}-\bar{y}\right)}^{2}}{n-2}}$, where $\bar{x}$ and $\bar{y}$ are the averages of variables *x* and *y*. The uncertainty region is shown as a continuous surface within the plots by connecting the edges of the pointwise estimates.

We examined whether single-gene mutations in *scn1lab* and *disc1* have an effect on group aggregation and alignment phenotypes measured at 7 dpf and at 21 dpf (Fig. 2). We used groups of five zebrafish of the same mutant type, which we denote by j. Let G_j_ denote the mutation status of group j, where G_j_ = 0 for a wild-type group and G_j_ = 1 for a mutant group, and assume a completely randomized assignment of the group mutation status. For example, G_j_ = 0 for a group of five *scn1lab*_allele2_^+/+^ fish and G_j_ = 1 for a group of five *scn1lab*_allele2_^+/−^ fish. For each group j, we measure an outcome Y_j_, such as group aggregation or group alignment. We denote Y_j_(G_j_ = 0) the potential outcome had group j been wild type and Y_j_(G_j_ = 1) the potential outcome had group j been mutant. We test the sharp null hypothesis stating that, for each group j, the potential outcomes are equal, that is, Y_j_(G_j_ = 0) = Y_j_(G_j_ = 1). We use T_Welch_ as our test statistic. For the aggregation phenotype, we calculated one-sided Fisher *P* values capitalizing on the results from (*24*); for the alignment phenotype, we calculated two-sided Fisher *P* values.

Using individual 7-dpf zebrafish in a cdOMR assay (Fig. 3), we also examined whether single-gene mutations in *scn1lab* and *disc1* have an effect on alignment. Here, we denote an experimental zebrafish unit by “i” and its mutation status by G_i_, where G_i_ = 0 for wild type and G_i_ = 1 for mutant, and assume a completely randomized assignment of the individual zebrafish mutation status. For each zebrafish i, we measure an outcome Y_i_, such as probability correct, interbout interval, or probability to turn in the same direction. We denote Y_i_(G_i_ = 0) the potential outcome had zebrafish i been wild type and Y_i_(G_i_ = 1) the potential outcome had zebrafish i been mutant. We test the sharp null hypothesis stating that, for each zebrafish i, the potential outcomes are equal, that is, Y_i_(G_i_ = 0) = Y_i_(G_i_ = 1). We chose T_Welch_ as our test statistic and calculated two-sided Fisher *P* values (or approximated ones based on 100,000 randomized allocations). Using this method, we compared the probability of correct turns, the interbout interval, and the integral of estimated turning distribution (turn angles of >20°) for 0, 25, 50, and 100% coherence levels. In addition, we also compared the probability of the fish to turn in the same direction 0.125 to 1.375 s after a swim bout during 0% coherent motion (Fig. 3D). For each of these measured phenotypes, we estimated two-sided Fisher *P* values as described above.

To report a measure of effect size in Figs. 1 and 2, we chose the commonly used Cohen’s *d* (*56*). In the case of a two-sample statistical model, $d=\frac{\left({\bar{x}}_{1}-{\bar{x}}_{2}\right)}{S_{p}}$, where ${\bar{x}}_{1}\;\text{and}\;{\bar{x}}_{2}$ are the means of the two groups, and *S_p_* is the pooled estimate of the SD of the two groups. In the case of a one-sample statistical model, we used $d=\frac{\left({\bar{x}}_{1}-\mu_{0}\right)}{S_{1}}$, where μ_0_ is the estimate of the mean of null distribution or the estimated baseline that we compared to. None of the reported *P* values and CIs were adjusted for multiple comparisons.

### Motion assay

The assay has been described previously (*39*). In brief, 7-dpf larvae were placed in custom-designed acrylic dishes (12 cm diameter, 5 cm height, black rim, and transparent bottom). The scene is illuminated from below with infrared light-emitting diode panels (940 nm, Cop Security). The fish are tracked with a camera (Grasshopper 3), a zoom lens (Zoom 7000, 18 to 108 mm, Navitar), and a long-pass filter (R72, Hoya). Posture analysis is performed in real time using custom-written software using Python 3.7 and OpenCV 4.1. Stimuli were presented from below (Aaxa P300 Pico Projector) onto mildly scattering parchment paper and consisted of ~1000 small (2-mm) white dots on a black background. We showed 0% coherence as a baseline stimulus for 5 s and then switched to (25, 50, or 100%) coherent motion at a constant speed (1.8 cm/s) for 10 s. Motion either went rightward or leftward relative to the body orientation of the fish. Each dot persisted for only 200 ms on average and stochastically disappeared and reappeared at a new location, so as to prevent fish from tracking individual dots. Following the coherent stimulus, the stimulus reverted to 0% coherence baseline.

### Genotyping

For group assays (Figs. 1 and 2 and fig. S1), fish were genotyped at 2 to 3 dpf using Zebrafish Embryonic Genotyper (wFluidx) or fin clipping and high-resolution melt analysis (HRM; primer sequences in table S1). Following all experiments, genotypes are confirmed by HRM following DNA extraction using hot shot genomic DNA preparation. Briefly, whole larvae are dissolved in 25 μl of alkaline solution (25 nM NaOH and 0.2 mM Na_2_ EDTA) for 1 hour at 95°C, and an equal volume of neutralizing solution (40 mM tris-HCl) is added afterward. The genomic preparation is diluted 1:20 before HRM.

### Drift-diffusion model

To better understand the origin of the observed behavioral phenotypes during motion integration, we use computational modeling. This approach provides us with a more detailed characterization of the behavior and allows us to indirectly infer how a mutation affects specific components of the sensory-motor transformation algorithm.

Previous work indicated that a simple DDM with a decision threshold can explain many aspects of the responses to the dot motion stimulus [Fig. 3E and fig. S3, A and B; (*39*, *40*)]. The model is based on the temporal integration of noisy [drawn from a Gaussian distribution *N*(0, σ)] motion evidence with certain coherence levels *C*(*t*). The integrator is leaky, and therefore, its signal *X*(*t*) increases slowly with a time constant (τ) when the motion stimulus starts and decays with the same dynamics when it stops

$$\tau \cdot \frac{\mathrm{dX}(t)}{\mathrm{dt}}=C(t)-X(t)+N(0,\sigma )$$

We solved this equation by using Euler’s method

τ·ΔX/Δt=C(t)−X(t)+N(0,σ)

ΔX=(C(t)−X(t)+N(0,σ))·Δt/τ

X(t+1)=X(t)+ΔX

Notably, we chose the same *dt* = 0.01 in all our model simulations (also see the agent-based model below). This was important as the noise term *N*(0, σ) in our stochastic differential equation would have a different influence on our results, depending on the choice of *dt*. This problem can be resolved by scaling σ by $\sqrt{\mathrm{dt}}$, which we did not do in our simulations. Therefore, the values of σ displayed in fig. S4B should be interpreted in the context of *dt*. In our model, fish swim spontaneously with two different probabilities. When the integrated motion evidence *X*(*t*) is below the decision threshold (T), animals swim forward with a probability of *p*_below_. When it is above the threshold, they make a turn with a probability of *p*_above_. The exact values of these probabilities indicate whether fish will initiate a swim event within a time step *dt* of our stochastic differential equation stimulation or not. In case of such events, forward swim heading angle changes were drawn from a Gaussian distribution *N*(0,5), while turning angle changes were drawn from a Gaussian distribution *N*( ±22,25), following the general shape of the measured heading angle change distributions (fig. S2C). Notably, none of the underlying five model parameters can be measured directly through behavioral experiments, requiring us to resort to an indirect method, a multiobjective fitting strategy.

### Multiobjective fitting algorithm

To uncover latent changes within the motion-integrating and decision-making circuits, we modified an evolutionary multiobjective optimization technique that can find the same global minimum and the same parameter set over repeated optimization runs (fig. S3C) (*57*, *58*). This approach has been used in the past to solve highly nonlinear models that require multiple behavioral features to be optimized simultaneously (*59*, *60*). One starts with a population of randomly chosen parameter sets (800 individuals in our case). Each parameter set gets evaluated, producing five behavioral features: (i) the probability to turn in the correct direction as a function of coherence (Fig. 3B), (ii) the interbout interval as a function of coherence (Fig. 3C), (iii) the probability to turn in the same direction for consecutive swims during 0% coherence (Fig. 3D), (iv) the binned probability to turn in the correct direction as a function of time and coherence (fig. S2B), and (v) the turn angle probability distribution (fig. S2, C and D). For features (i) to (iv), the algorithm computes the mean squared error (MSE) between model simulation results and experimental data. For feature (v), we determined the distance between model and experiment using a Gaussian mixture model approach: We first fitted each probability distribution with three overlapping Gaussian functions (one for left turns, one for right turns, and one for forward swims). We then computed the MSE between the weights (peak heights) of the three resulting Gaussian functions. We also performed model optimizations with alternative distance metrics, such as the mean squared logarithmic error, leading to comparable results. Using these five distance functions, the multiobjective algorithm then chooses which individuals are mutated and which ones will exchange parameter information, using crossover, to build the next generation.

We first sought to test that the used multiobjective optimization algorithm, a Python-based open-source package called pymoo (version 0.4), works as expected. To this end, we inspected the evolution of Pareto fronts during the optimization procedure. We found that all tested pairs of distances approached the origin (0, 0) of the coordinate system and that the distribution of individuals covered increasingly more space of the error landscape over generations. We then tested the algorithm on artificially created surrogate datasets (fig. S3, D to H), where we explicitly selected certain hidden variables. The extent of their successful recovery allows for a quantitative evaluation of the multiobjective optimization technique. Using a set of parameters closely following our recently hand-tuned parameter set (*39*), we find that the five extracted behavioral features generally capture what we find in the experiment (compare Fig. 3, B to D, and figs. S2, B and C, and S3D). After a few generations, the optimization algorithm was able to identify individuals that had near-zero error in at least one of the behavioral features. Last, to assign a compromise error value to each individual, we computed a weighted sum of the five normalized distance functions. To correct for the fact that each distance function has its own scale and unit, we first normalized values using the 75th percentile of the distribution of error values, which brought all distributions into a comparable range. As we consider the interbout interval as the most important behavioral feature that our model should definitely capture, we next multiplied the weight for the interbout interval distance function by 3; for all other distance functions, we used a weight of 1.

It took about 80 generations for this compromise error function to converge (fig. S3E). Notably, our algorithm does not find individuals with exactly zero error. This is expected as our simulation is stochastic and therefore does not produce the exact same behavior in every stimulation run. We repeated the optimization algorithm 12 times for four models with different parameter sets (fig. S3F). For each of the runs and models, we find that the algorithm successfully reduces the five error functions as well as the compromise error. At the end of each optimization run, we then picked the one individual with the smallest compromise error value and compared its parameter values to the parameters originally used to create the surrogate dataset (fig. S3G). We find that our algorithm can reveal these values and that repeated optimization runs produce more or less the same results. Looking at one of those optimized models, we confirm that the behavioral features do closely resemble the ones from the original dataset (fig. S3H). In summary, we conclude that our multiobjective evolutionary optimization is capable of extracting the hidden variables in our motion integration and decision-making model.

### DDM parameters extracted from motion integration assay

Knowing that our multiobjective fitting algorithm can reveal the latent variables in our DDM, we next applied this strategy to real experimental behavioral data of larval fish with different genotypes. For all tested genotypes, the optimization algorithm produces behavioral dynamics closely mimicking the ones found in experiments (compare Fig. 3, B to D and F to H, and fig. S2, B to D and F to H). We repeated the optimization algorithm 12 times for each dataset. We find solutions to have similarly small error values (fig. S4A) and that the estimated model parameters are more or less identical after each run (fig. S4B).

### Collective behavior model

We simulated groups of five fish freely swimming in a circular arena. The arena was modeled with a diameter of 1024 pixels. Each fish had a random starting position (*x* and *y*) and orientation in the arena and length of fishsize = 20 pixels. At every time point during the simulation, fish made swimming decisions based on two simple sensory-motor transformation rules: the tendency to avoid or approach clutter and the tendency to align with global motion drift.

To compute clutter, we determined how much space another fish (fish *i*) occupies in the visual field of the focal fish (fish *j*) and added all values from the right hemisphere and subtracted all values from the left hemisphere

$$\text{Perceived clutte}r_{j}(t)=\sum_{i\neq j}^{N}\text{hemispher}e_{\text{weight}}\cdot 2\cdot \text{arctan}(\text{fishsize}/2\cdot d_{i,j})$$

where $d_{i,j}=\sqrt{(}{\left(x_{i}-x_{j}\right)}^{2}+{\left(y_{i}-y_{j}\right)}^{2})$ is the Euclidean distance between animal pairs and where hemisphere_weight_ was either +1 or −1, depending on whether fish *i* was in the right or left hemisphere relative to the orientation of fish *j*. *N* is the number of fish (= 5 fish in our simulations). Hence, the sign and amplitude of the resulting signal reflect the asymmetry perceived by the focal fish.

To compute the perceived motion force [perceived motion*_j_*(*t*)] by the focal fish (fish *j*), we first performed projections of the motion vectors of all other fish (fish *i*) onto its circular field of view (fig. S5, A and B). As the vector length of the fish *i* is proportional to its momentary swim speed *v*(*t*), fast-swimming fish will produce a larger projection arc than slowly swimming fish. Notably, if fish *i* moves in the front or in the back of the focal fish (fish *j*), even when it moves in the same direction, this type of radial projection will produce different signs, which is not realistic. For example, when the other fish is in the front, moving left, the sign will be positive (projection arc is counterclockwise). When it is in the back, moving left, the sign will be negative (projection arc is clockwise). To correct this problem, we sign-inverted all projections where the other fish *i* started in the back of the focal fish *j*. Last, we multiplied these projections with a weight, depending on where on the retina the image is projected (fig. S5C). Our projections guarantee four important properties: (i) If the other fish approaches the focal fish directly—resembling a looming stimulus—or if it radially recedes, we will not obtain a motion force. This is biologically plausible because such stimuli would not specifically activate circuits tuned to object or global motion (fig. S5A). (ii) Motion force transitions between the back and the front are smooth. This is important as, otherwise, moving objects crossing this line would produce a sudden discontinuity in the motion force. (iii) Fish moving in parallel to the focal fish will not produce turning forces (fig. S5A). (iv) Movements in the distance will produce smaller motion forces than proximal ones.

To obtain the total momentary perceived evidence for fish *j*, we simply added the values for clutter and motion

$${\text{Sensory evidence}}_{j}(t)=w_{\text{clutter}}\cdot f_{\text{clutter}}\cdot {\text{perceived clutter}}_{j}(t)+w_{\text{motion}}\cdot {\text{perceived motion}}_{j}(t)$$

where *w*_clutter_ is the weight of the clutter system, *f*_clutter_ is the genotype- and age-dependent factor of the clutter system, and *w*_motion_ is the weight of the motion system.

Following our DDM (Fig. 3E), we integrated this value over time. Accordingly, when the integrated value was between the positive and negative thresholds, animals swam forward; when it was above the positive threshold, they turned to the right, and when it was below the negative threshold, they turned to the left. When swimming forward, animals covered 20 pixels per bout (about one body length) along their momentary axis of orientation. Moreover, animals stochastically changed their body orientation by a bit. Following the experimentally measured heading angle change distributions (fig. S2C), we drew those angles from a Gaussian distribution centered at 0° with an SD of 5°. Similarly, for initiating right or left turns, we drew angles from a Gaussian distribution centered at 22° or −22°, respectively, with an SD of 25° (fig. S2C). To capture the length and short gliding phase of forward swims and turns, we applied a low-pass filter with a time constant of 100 ms to these events, resulting in bout-like animal movement.

Notably, *w*_clutter_ and *w*_motion_ are the only free parameters of our model, and all other parameters are directly extracted from experimental data. Although tweaking those values would likely have resulted in overall improved model performance, we wanted to work with the most minimal model and, therefore, simply set both weights to 1. We further chose *f*_clutter_ for the 7-dpf *scn1lab_allele2_^+/+^* wild type to be −1, which reproduced the weak avoidance and alignment of 7-dpf *scn1lab_allele2_^+/+^* wild-type larvae. The values for *f*_clutter_ for the other genotypes and ages were then scaled according to the experimentally measured probability to turn toward clutter (Fig. 2, E and F, and fig. S1E). For example, the clutter response strength for the 7-dpf *scn1lab_allele2_^+/−^* was twice as strong as the one for the 7-dpf *scn1lab_allele2_^+/+^.* Hence, for this genotype, we scaled *f*_clutter_ to −2. The clutter response of 21-dpf *scn1lab_allele2_^+/+^* fish was positive and about three times as strong in amplitude as the one for 7-dpf *scn1lab_allele2_^+/−^* larvae. Hence, we scaled this value to +3. This procedure led to the following clutter response factors for the 7-dpf animals: *scn1lab_allele2_^+/+^*: −1; *scn1lab_allele2_^+/−^*: −2; *scn1lab_allele1_^+/+^*: −1; *scn1lab_allele1_^+/+^*: −1.5; *disc^+/+^*: −2; *disc^−/−^*: −1.5. For the 21-dpf animals, we obtained the following: *scn1lab_allele2_^+/+^*: +3; *scn1lab_allele2_^+/−^*: +3; *scn1lab_allele1_^+/+^*: +3; *scn1lab_allele1_^+/+^*: +2; *disc^+/+^*: +1; *disc^−/−^*: +2.

For all other parameters of our model (time constant, τ; noise, σ; decision threshold, **T**; swim probabilities below and above the threshold, ***p***_**below**_ and ***p***_**above**_), we chose exactly the results obtained from the multiobjective fitting procedure (fig. S4B). For testing the clutter or motion systems in isolation (Fig. 4, C and D), we simply set the weight of the respective other system to zero. Every time when an animal reached the circular border of the arena, we picked a new random orientation vector. We did not reflect or wrap trajectories across the border. We simulated the collective behavior model for 600 s with a time step of *dt* = 0.01 using the forward Euler method (see DDM above). We used Python 3.8 and the real-time compiler Numba. We stored the resulting trajectories in the exact same format as used for our experimental data, allowing us to use the same analysis scripts to extract values for aggregation and alignment (Fig. 1). The simulation source code is available online:

https://github.com/arminbahl/mutant_zebrafish_behavior.

## Notes

View/request a protocol for this paper from *Bio-protocol*.
