# Supplementary material for: Collective behavior emerges from genetically controlled simple behavioral motifs in zebrafish
Source: Sci Adv. 2021 Oct 6;7(41):eabi7460. doi: 10.1126/sciadv.abi7460 (PMC8494438; doi:10.1126/sciadv.abi7460)
Supplement: Supplementary file 1 — Table S1 Figs. S1 to S5 [file sciadv.abi7460_sm.pdf]

Science Advances
AAAS

Supplementary Materials for

Collective behavior emerges from genetically controlled simple behavioral motifs in zebrafish

Roy Harpaz, Ariel C. Aspiras, Sydney Chambule, Sierra Tseng, Marie-Abèle Bind, Florian Engert, Mark C. Fishman*, Armin Bahl

*Corresponding author. Email: mark_fishman@harvard.edu

Published 6 October 2021, *Sci. Adv.* **7**, eabi7460 (2021)
DOI: 10.1126/sciadv.abi7460

The PDF file includes:

Table S1
Figs. S1 to S5

Other Supplementary Material for this manuscript includes the following:

Movies S1 to S3

Gene	Primer1	Primer2
scn1lab $_{allele1}$ (*scn1lab*$_{nv978}$)	AAACTCTTCCTCTTTCTGTTGAGC	GTGCAGCTGGAAAGCCCTAT
scn1lab $_{allele2}$ (*scn1lab*$_{sa16474}$)	GCCTGTGCTGTGATTGGTTG	CGAACTCAGTCTAGACCCCC
disc1$_{nv1142}$	GCTCAACAGGTGGTGTGAAT	AGCTGGTTAATCGGCTCAGA

Table S1: Genotyping Primers.

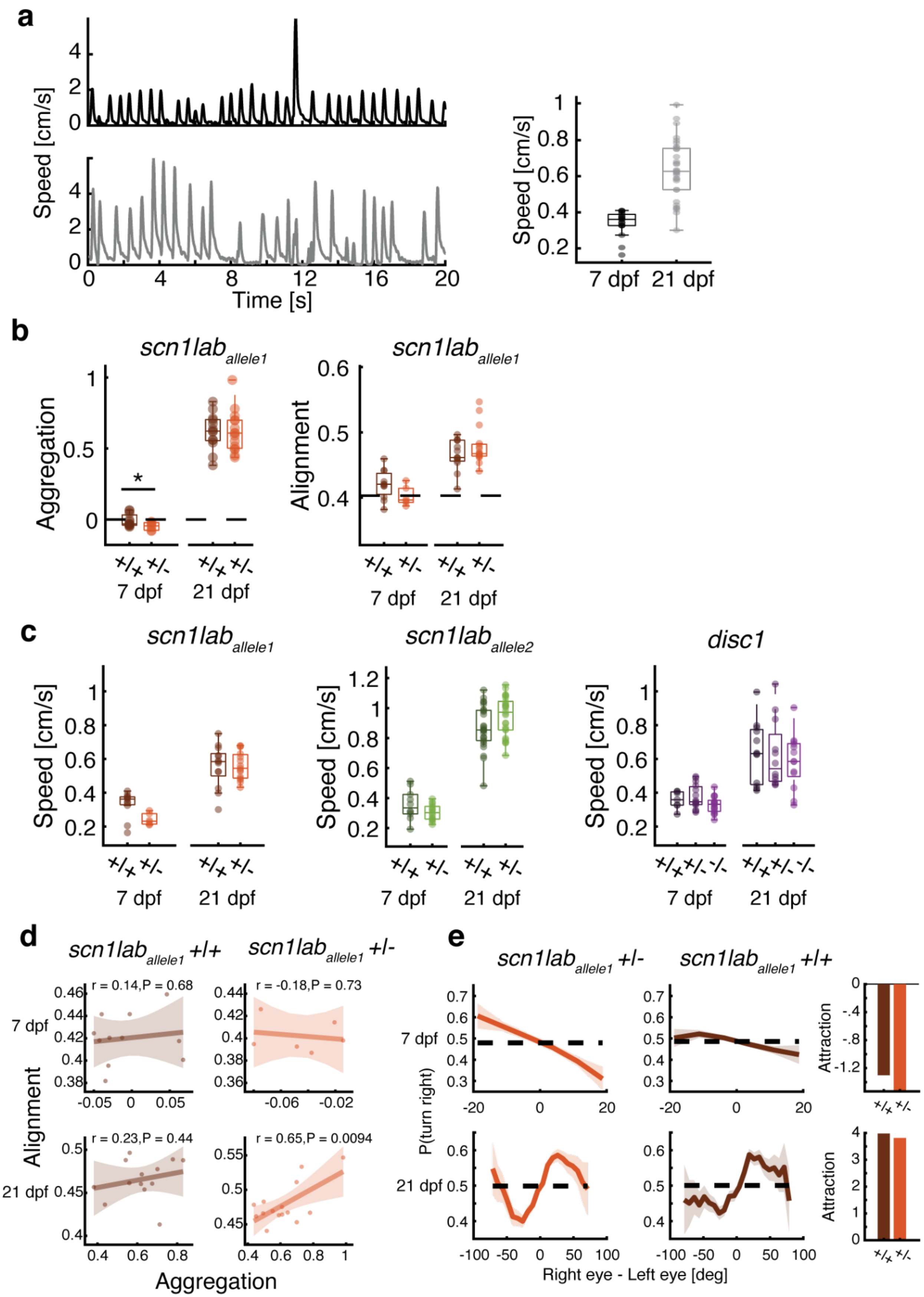

a
Speed [cm/s]
Time [s]
7 dpf
21 dpf
b
scn1lab allele1
Aggregation
Alignment
+/+
+/-
*
c
scn1lab allele1
scn1lab allele2
disc1
-/-
d
scn1lab allele1 +/+
scn1lab allele1 +/-
r = 0.14, P = 0.68
r = -0.18, P = 0.73
r = 0.23, P = 0.44
r = 0.65, P = 0.0094
Aggregation
e
P(turn right)
Right eye - Left eye [deg]
Attraction

Figure S1 | Single gene mutations do not affect speed during group assay. (a) Larval and juvenile zebrafish swim in bouts, with an overall increase in speed for older animals. (**b**) A mutation in the *scn1lab* gene (allele1) leads to more dispersed groups, only in 7 dpf larvae ($P_{Fisher} \approx 0.03$, $N_{+/+} = 11$, $N_{+/-}=6$ groups) and has no effect on group alignment. (**c**) Our data did not allow us to report a speed difference between mutant and wild-type animals, but speed is always larger in older animals. **(d)** Correlation of alignment and aggregation in $scn1lab_{allele1}$ 7 (top row) and 21 dpf (bottom row) fish. Our data did not allow us to report a correlation between alignment and aggregation at 7 dpf (top), while 21 dpf Mutant $scn1lab_{allele2}^{+/-}$ fish (bottom right) show a positive correlation, similar to the results seen in $scn1lab_{allele1}^{+/-}$ (**Fig. 1g**). **(e)** Left: At 7 dpf $scn1lab_{allele1}^{+/-}$ fish show an increase in the tendency to turn away from visual clutter, whereas at 21 dpf $scn1lab_{allele2}^{+/-}$ fish show a reduced tendency to turn towards high visual clutter. Right: The integral of the curves on the left symmetrized such that repulsion from clutter is negative and attraction is positive.

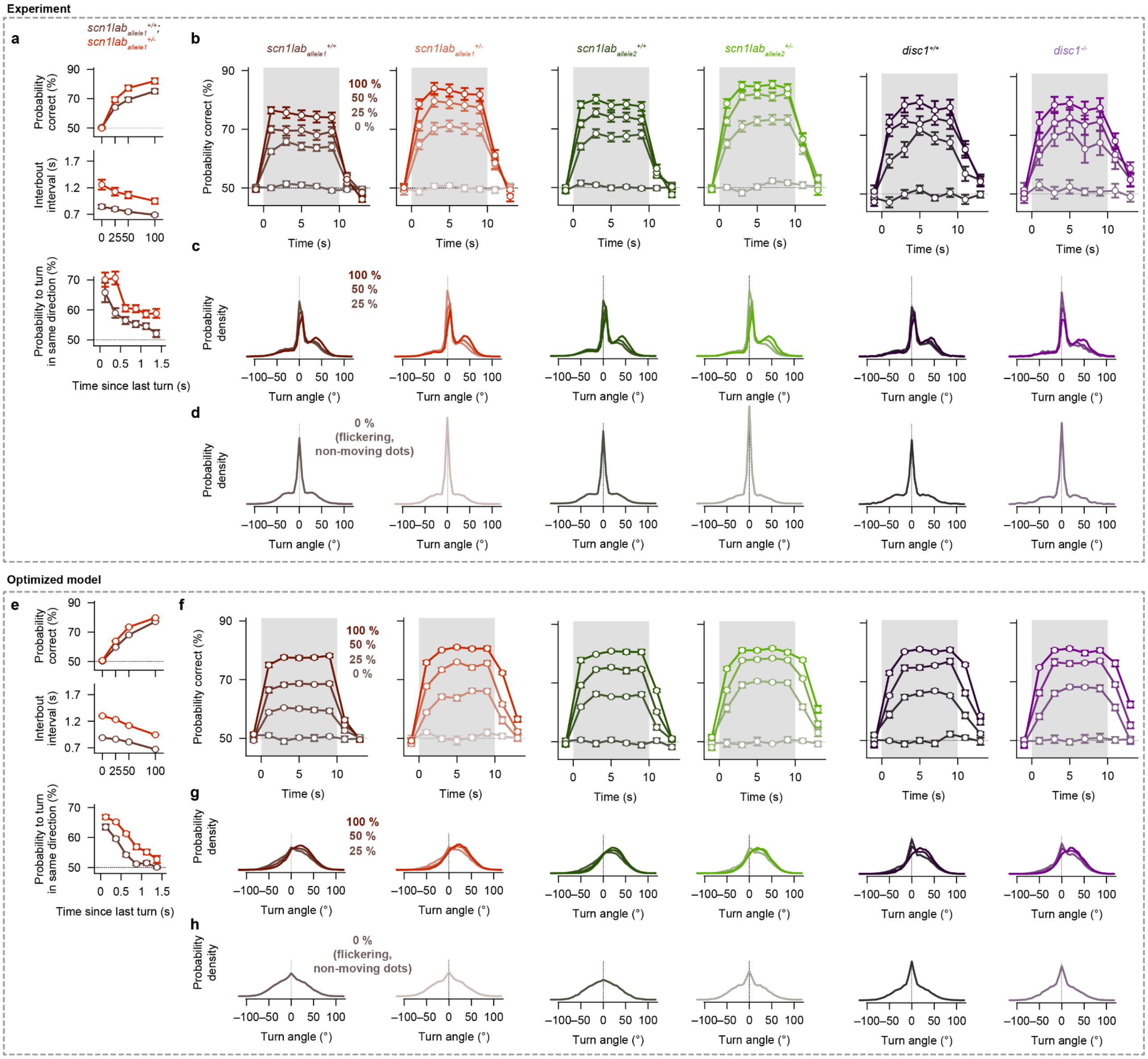

Figure S2 | More detailed behavioral features and modeling results for the random dot motion integration assay for 7 dpf larvae. (a) The same analysis of experimental data as in **Fig. 2b–d** but for *scn1lab*$_{allele1}$ mutant fish. The phenotypes of a mutation in this allele quantitatively match the ones for *scn1lab*$_{allele2}$. (**b**) Probability correct over time for different coherence levels shows that both *scn1lab*$^{+/-}$ mutants have an increased average probability correct and slower integration dynamics compared to *scn1lab*$^{+/+}$ sibling controls. For *disc1*$^{-/-}$ mutants, the response dynamics relative to *disc1*$^{+/+}$ sibling controls does not change. (**c**) Turning angle probability density distribution shows that *scn1lab*$^{+/-}$ mutant animals have a slightly higher tendency to make turns (30°-60°) than wild-type control siblings. (**d**) Turning angle probability density distributions for 0 % coherence (non-moving) dots for mutant animals and their sibling controls. (**e–h**) Same analyses as in (**a–d**) but for the optimized models (same models as in **Fig. 2f–h**). In general, the model captures the phenotypes for *scn1lab*$_{allele2}$ *and* the overall dynamics of the probability correct as a function of time. It also qualitatively reproduces the turning angle probability density distributions. Color saturation indicates coherence level (from less saturated to more saturated: 0%, 25%, 50%, and 100%). Darker colors indicate sibling controls. Red and green lines are *scn1lab*$^{+/-}$ fish, respectively. Violett lines indicate *disc1*$^{-/-}$ fish. N = 34, 27, 44, 36, 21, and 16 fish for genotypes *scn1lab*$_{allele1}^{+/+}$, *scn1lab*$_{allele1}^{+/-}$, *scn1lab*$_{allele2}^{+/+}$, *scn1lab*$_{allele2}^{+/-}$, *disc1*$^{+/+}$, *and disc1*$^{-/-}$, respectively, in **a–d**. N = 12 models (different optimization repeats) for each genotype in (**e–h**). All error bars are ±sem. Same fish and models

as in **Fig. 2b–d,f–h**.

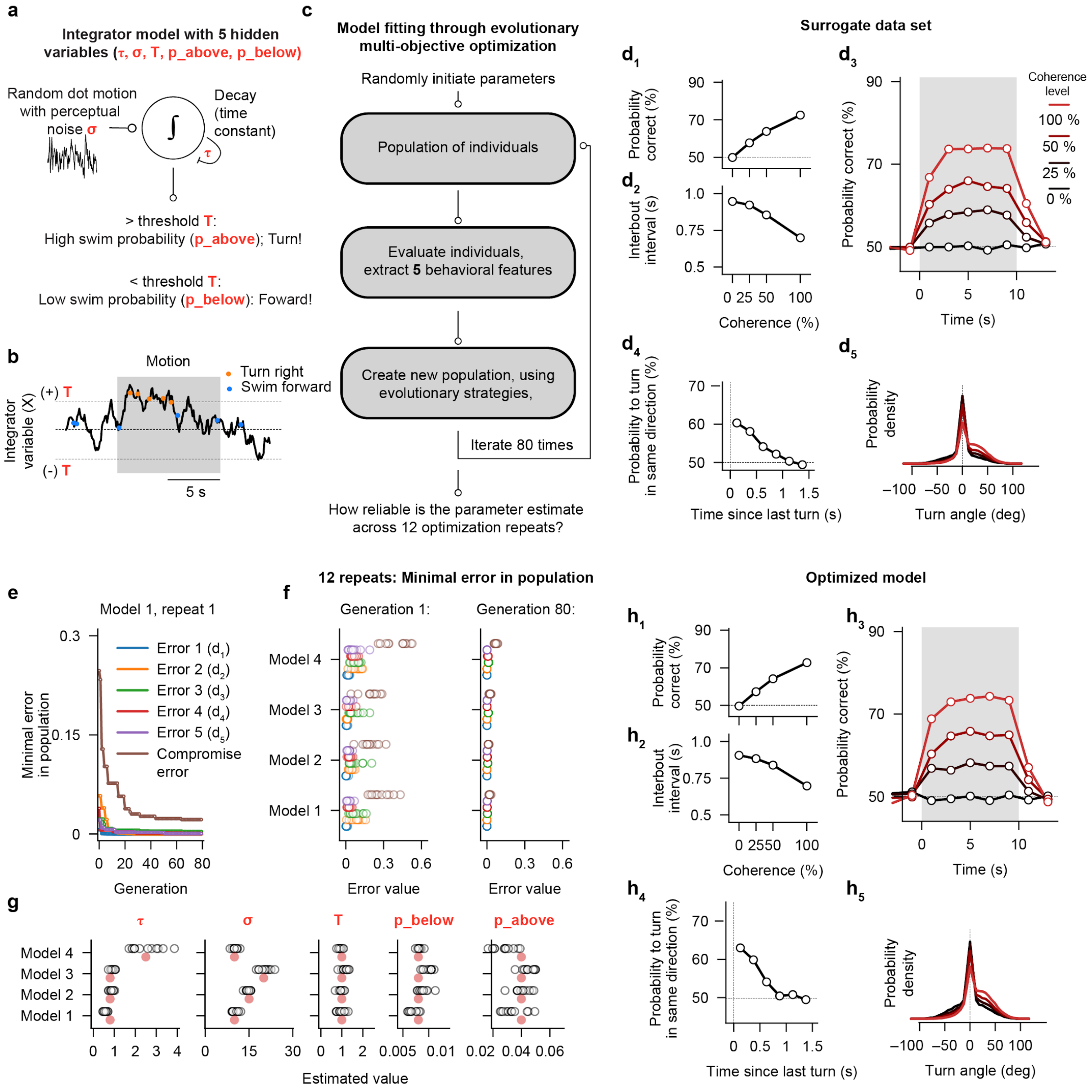

Figure S3 | Multi-objective optimization strategy on surrogate data. (**a**,**b**) Schematic of the integrator and decision-making model. Motion evidence with perceptual noise (σ) is integrated by a leaky integrator with a time constant (τ). When the integrated value is below the decision threshold (T), the model creates forward swims with a probability of p_{below} (blue dots). Otherwise, it makes turns (orange dots) with a probability of p_{above}. (**c**) Flow diagram of the evolutionary multi-objective optimization strategy. The algorithm starts with a population of randomly chosen individuals (parameter sets) and uses evolutionary principles to iteratively propagate models across generations. Models are chosen based on 5 multi-objective behavioral features, without needing to weigh them. We determine the general reliability and quality of the fitting algorithm by trying to uncover a few hidden model parameter combinations used to create artificial surrogate datasets. (**d_1–d_5**) The five behavioral features used as error functions during the multi-objective optimization (same analysis as in **Fig. 2b–d** and **Fig. S2b,c**). These example traces were created using the manually chosen parameter set given by the red dots for model 1 in panel **f**. (**e**) Evolution of error over generations for the 5 error functions. After a few generations, these error values converge to nearly zero. The compromise error (a

range-corrected weighted sum of all 5 error functions, see methods) requires more generations to converge. (**f**) Minimal error for the 5 error functions and the compromise error for the first and last generation for 12 optimization repeats and 4 example surrogate data sets. In the last generation, all 12 optimization repeats lead to almost identical error values. (**g**) Estimated parameters for the 4 example models. Optimization repeats are indicated with open black circles. The target parameter used to create the surrogate dataset is indicated with the red circle. The optimization algorithm can reliably reveal the hidden variables. (**h_1–h_5**) Simulation results of the optimized model following parameter optimization using the surrogate dataset of model 1 as a target (**d_1–d_5**). The objectives between the two simulations precisely match.

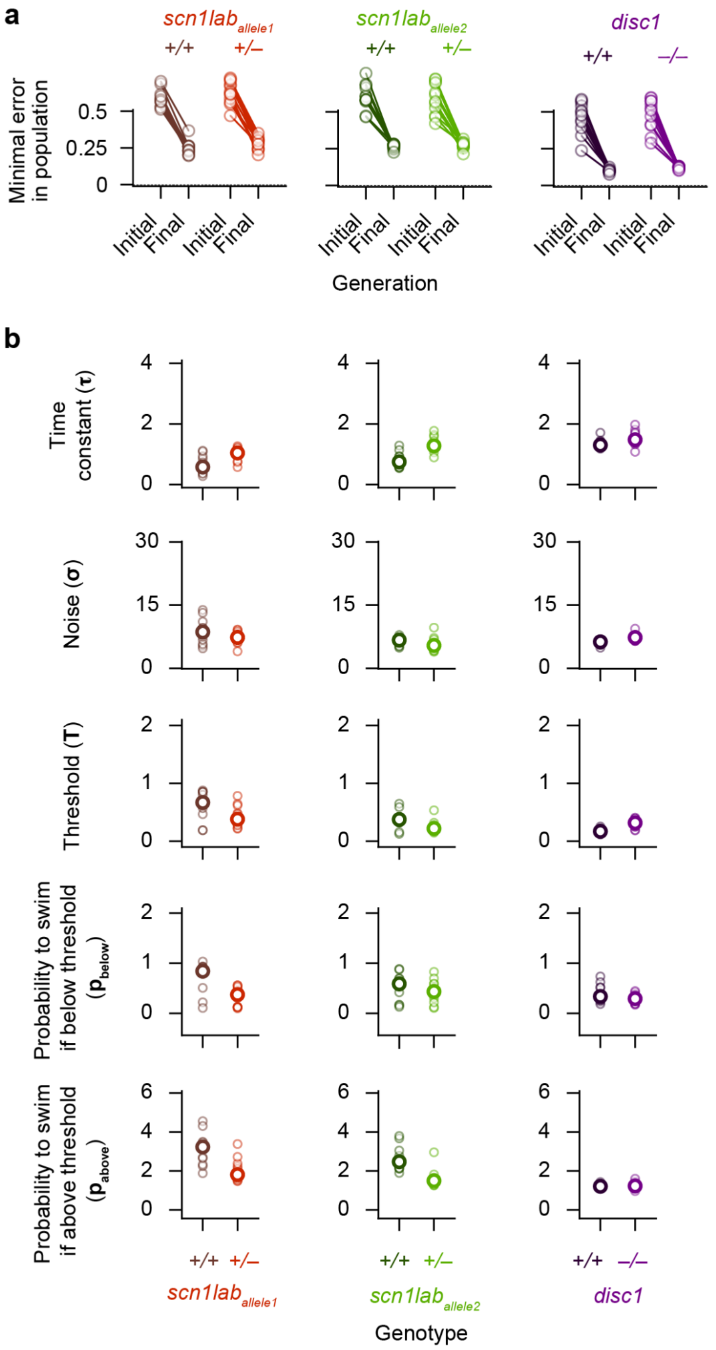

Figure S4 | Mutant larval zebrafish have specific algorithmic alterations in their ability to integrate motion signals. (a) Value of the compromise error function of the best individual in the initial generation and after 80 generations. 12 optimization runs and 6 target genotypes (3 mutants with respective sibling controls). **(b)** Estimated model parameter values of the best individual for each genotype and optimization run.; N = 12 model optimization repeats. Small open circles are individual repeats, bigger open circles are median parameter values.

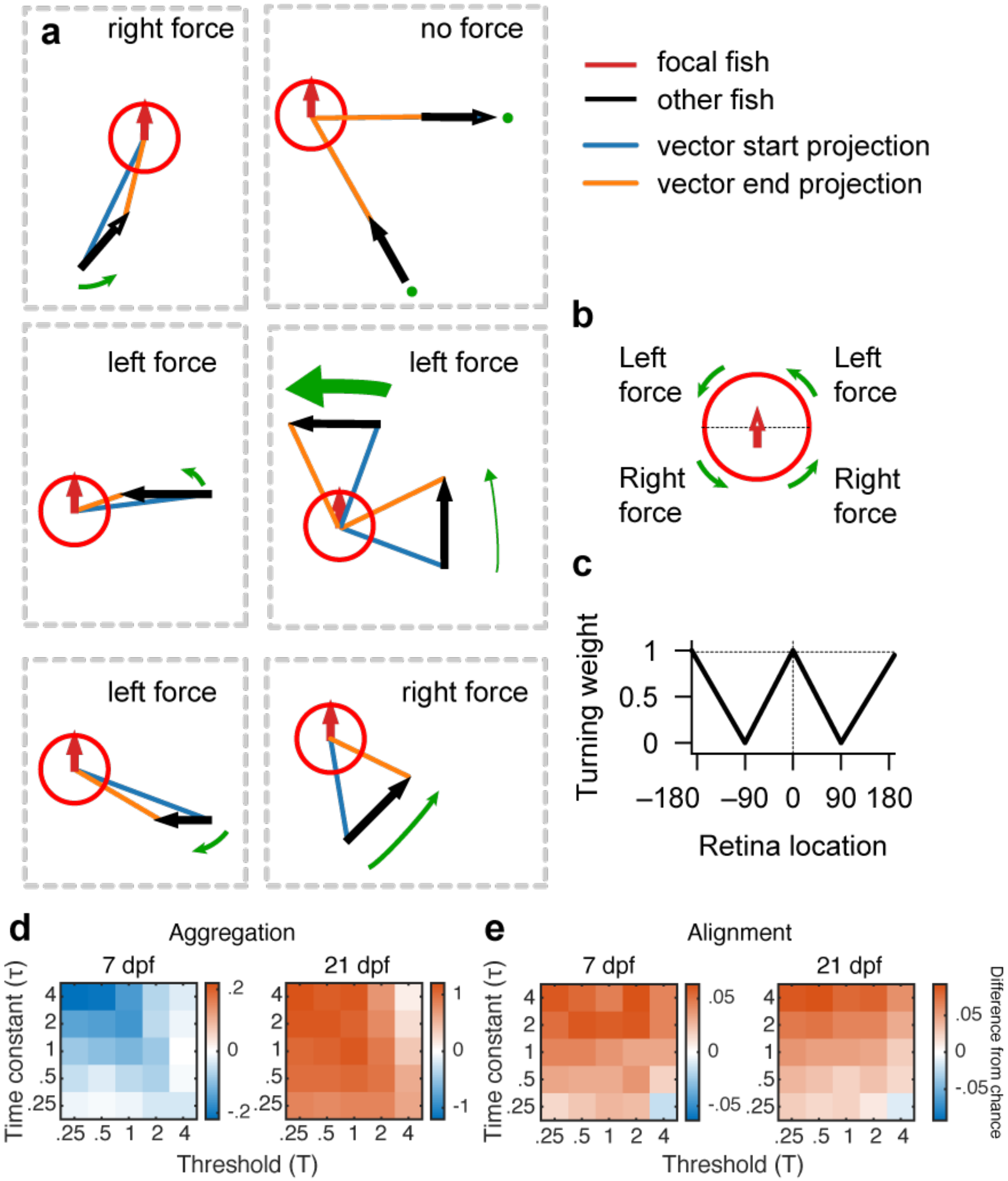

Figure S5 | Retinal motion projections and parameter robustness of our agent-based model. (a–b) Depiction of example turning "forces" generated by a fish moving in the vicinity. Turning forces are determined by first computing the angular size of the protection of the movement of the other fish. We then multiply the strength of this force with a turning weight, as a function of retinal position (**c**). **(d,e)** Model performance represented as average group aggregation (d) and alignment (e). for systematic variations of the parameters threshold (T) and time constant (τ). We started with the original parameters (**Fig. S4**) from our agent-based model (**Fig. 4**) and multiplied them with 5 different scaling factors (1 indicates that the parameter is not scaled). Values are shown as differences from shuffled control groups (see methods). Group structure in most pronounced for large time constants (τ) and small threshold (T) values.
